# Medical students’ perceptions of learning modalities: development and psychometric validation of the e-learning and face-to-face learning experience questionnaire

**DOI:** 10.1038/s41598-026-58815-1

**Published:** 2026-06-19

**Authors:** Zahra Karimian, Navab Momeni, Hamid Mohammadi, Maryam Jalali

**Affiliations:** 1 Department of e-Learning in Medical Sciences, Virtual School, Center of Excellence in e-Learning, Shiraz University of Medical Sciences, Shiraz, Islamic Republic of Iran; 2 School of Medicine, Shiraz University of Medical Sciences, Shiraz, Islamic Republic of Iran; 3 Department of Pediatrics, School of Medicine, Neonatal Research Center, Namazi Teaching Hospital, Shiraz University of Medical Sciences, Shiraz, Islamic Republic of Iran

**Keywords:** Learning Modalities, Student Engagement, Peer assisted learning, Educational Technology, Distance education, Medical Education, COVID-19 Pandemic, Education, Health care, Psychology, Psychology

## Abstract

Amidst the growing demand for effective blended learning, particularly post-COVID-19, understanding the key factors influencing student learning experiences is paramount. This study addresses this need by designing and validating a comprehensive questionnaire (ELFEQ) to assess these factors across both face-to-face (F2F) and e-learning environments in medical education. A quantitative, cross-sectional study was conducted with 298 students from Shiraz University of Medical Sciences (SUMS), Iran. Data were collected using the researcher-developed 27-item ELFEQ questionnaire (rated on a 5-point Likert scale). Exploratory Factor Analysis (EFA) with Varimax rotation was used for initial factor extraction, and Confirmatory Factor Analysis (CFA) was subsequently performed. Reliability was assessed using Cronbach’s alpha. Data analysis was performed using SPSS Version 24. The analysis revealed six primary factors influencing student learning experiences, including Peer Interaction, Teacher-Student Interaction, Examination and Assessment methods, Emotional Comfort, Content Quality, and Assignments. The highest factor loading was observed for peer interaction and collaborative learning. The questionnaire accounted for approximately 70% of the ELFEQ construct, with a reliability coefficient of α = 0.933. The Content Validity Index (CVI) was 0.976, and the Content Validity Ratio (CVR) was 0.898. The Kaiser-Meyer-Olkin (KMO) measure yielded a value of 0.920, indicating excellent sampling adequacy, while Bartlett’s Test of Sphericity was significant (*P* < 0.001), confirming the suitability of the data for factor analysis. The findings suggest that effective educational practices must integrate diverse teaching methods and assessment strategies to accommodate various learning styles. Both F2F and online environments offer unique advantages that can be leveraged through a blended approach. The study underscores the importance of creating inclusive and supportive learning environments that prioritize student comfort and engagement, ultimately enhancing educational outcomes.

**Supplementary Information:**

The online version contains supplementary material available at https://doi.org/10.1038/s41598-026-58815-1.

## Introduction

The advancement of modern technologies, particularly following the COVID-19 pandemic, has significantly reshaped the educational landscape, compelling institutions worldwide to shift from traditional face-to-face (F2F) learning to online modalities^1^. This transition has revealed numerous challenges and disparities between these two forms of education, highlighting the need for a comprehensive evaluation of their effectiveness. Therefore, assessing the quality of e-learning in comparison with F2F instruction has become increasingly important.

Online learning has facilitated rapid advancements in medical education^2^ and has played a central role in teaching and learning since the onset of the pandemic^3^. Nevertheless, educators and students have encountered challenges such as technological barriers, reduced engagement, and limitations in assessment strategies^4–6^. For instance, students have reported difficulties in retaining information and engaging meaningfully with online course materials^7–9^.

Evidence regarding the comparative effectiveness of F2F and online learning remains inconclusive. Some studies suggest that F2F instruction is more effective than online learning^10–12^, whereas others report comparable or even superior outcomes for online learning in specific domains such as skill development^13–17^. For example, Siddiqui et al.^18^ reported that e-learning provides advantages such as flexibility and easy access to learning materials, whereas in-person instruction enhances clinical skills and social interaction. The authors concluded that e-learning should complement, rather than replace, traditional teaching methods.

Similarly, Kenzaka et al.^19^ compared learning outcomes between F2F and online formats in a clinical reasoning lecture and found comparable comprehension scores, despite higher satisfaction with audiovisual quality and accessibility in the online group. Li et al.^20^ reported that although dental students had positive perceptions of online learning, F2F learners demonstrated superior performance in attendance, group interaction, and overall proficiency. In line with these findings, Foo et al.^21^ showed that students in F2F tutorials achieved higher scores in key skill domains compared with their online counterparts.

Hollister et al.^12^ examined undergraduate students’ online learning experiences and reported reduced engagement and attendance in live lectures, with 72% of students indicating that low engagement negatively affected their learning experience. Students also reported difficulties in maintaining peer and instructor connections, as well as managing course workload. Despite these challenges, overall perceptions of instructional staff remained positive, and students reported greater comfort in participation within online environments.

Both F2F and electronic learning environments offer distinct advantages that may influence learning outcomes depending on context. E-learning tools such as discussion forums, quizzes, polls, and chat rooms facilitate interaction and content delivery while overcoming spatial and temporal constraints^15^. However, studies have also identified barriers including limited interaction, technical issues, and challenges in online assessment. Notably, it has been suggested that a substantial proportion of curricular content (approximately 30%) may continue to be effectively delivered through online formats post-pandemic^22^.

Given the mixed evidence and the ongoing integration of online learning into medical education, a comprehensive evaluation of these modalities is essential. Although several instruments have been developed to assess student perceptions of online and F2F learning, many are limited to non-medical contexts or fail to simultaneously evaluate both modalities within a single validated framework^18,23–26^. Moreover, the unique characteristics of medical education—particularly the integration of clinical training, practical skills, and patient interaction—necessitate a context-specific instrument^27,28^.

To address this gap and strengthen the conceptual foundation of the present study, three established pedagogical theories were adopted: the Community of Inquiry (CoI) framework^29^, Cognitive Load Theory^30^, and Self-Determination Theory (SDT)^31^. These frameworks guided the development of the questionnaire by ensuring coverage of key dimensions of the learning experience, including teaching presence, social presence, cognitive load, and learner motivation. Accordingly, this study aims to develop and psychometrically validate a questionnaire specifically designed for medical students to assess comparable learning experiences in both e-learning and F2F environments. The resulting instrument is intended to provide a reliable and context-appropriate tool to enhance educational quality and inform future improvements in medical education.

## Methods

### Study design

The present study was conducted using a cross-sectional psychometric design. It focused on medical students at Shiraz University of Medical Sciences (SUMS) during the 2021–2022 academic year.

### Participants and sampling

The statistical population included 8,000 undergraduate students enrolled in the 2021–2022 academic year. These students had completed at least two semesters both before and during the COVID-19 pandemic. This research focused on undergraduate students due to the uniformity of their learning environments and interactions with faculty, which were facilitated by similar platforms. In contrast, graduate students encountered unique conditions characterized by more self-directed learning, resulting in different requirements and dependencies on digital and in-person learning settings compared to their undergraduate counterparts. Participants were required to have the ability to compare face-to-face and e-Learning environments. Students entered the study voluntarily and could withdraw at any time. Those who did not respond to more than 20% of the questions were excluded from the study.

#### Consideration

In Iranian medical universities, undergraduate education includes both Bachelor’s students and professional doctoral programs (Medicine, Dentistry, and Pharmacy), who enter directly after high school and follow a structured curriculum. This group had relatively uniform exposure to the institutional Learning Management System (LMS) during the COVID-19 pandemic. The inclusion of this population was intended to reduce educational heterogeneity and enhance internal validity during the instrument development phase.

### Sample size

Since this study focused on instrument development and construct validity, Exploratory Factor Analysis (EFA) with Varimax rotation and Kaiser normalization was employed to identify the underlying factor structure. Furthermore, Confirmatory Factor Analysis (CFA) was conducted to verify the factor structure and evaluate model fit indices.

The sample size was determined based on a minimum ratio of ten participants per questionnaire item. Given that the questionnaire contained 27 items, a minimum of 270 fully completed responses was required. To ensure an adequate response rate, the questionnaire link was distributed via email to 350 students.

Data from completed questionnaires were recorded in Excel and stored in the survey system. Although participant confidentiality and anonymity were maintained, IP addresses were temporarily accessible for data management purposes. Any responses with identical entries submitted from the same IP address were screened to prevent duplication. Ultimately, no duplicate responses were identified.

### Sampling

Sampling was carried out using proportionate stratified random sampling based on faculty distribution. Within each faculty, participants were selected using a random sampling method. The student email lists, categorized by faculty, were obtained from the university’s Education Office, and 350 students were randomly selected in proportion to the size of each faculty population.

#### Inclusion/exclusion criteria

It should be noted that the inclusion criteria comprised undergraduate students (BSc and professional doctorate) who were enrolled at the university during the academic years 2021–2022 and used the university’s Learning Management System (LMS). These students had also experienced face-to-face learning. They were selected because the purpose of this study was to compare learning tools across both traditional and online learning environments. Postgraduate students were excluded due to differences in their educational structure, assignments, and interactions compared to undergraduate and professional doctorate students. In addition, questionnaires with more than 20% missing responses were excluded from the analysis.

### Instruments

The research instrument was a researcher-developed questionnaire, the E-Learning and F2F Learning Experience Questionnaire (ELFEQ), consisting of 27 items across six dimensions: peer interaction, instructor interaction, assessment, convenience, content, and assignments. Responses were measured using a 5-point Likert scale (Very High = 5, High = 4, Moderate = 3, Low = 2, and Very Low = 1).

The instrument was developed through qualitative methods involving focus groups with 15 virtual education coordinators from various faculties at Shiraz University of Medical Sciences (SUMS) over four online sessions.

Content validity was initially assessed using both face and content validity measures through the Content Validity Index (CVI) and Content Validity Ratio (CVR). Construct validity was evaluated based on data collected from 298 students. Reliability was assessed using internal consistency analysis with Cronbach’s alpha (see Appendix 1).

### Data collection

Data were collected electronically via an online questionnaire. The e-questionnaire was designed on the Porsline.ir platform, and links were sent to undergraduate students’ emails. Initially, a list of students and their emails was obtained from the educational vice-presidency, and emails were sent according to each faculty’s proportion. The questionnaire link was also distributed among students through social media.

The questionnaire was designed online, and the link to the questionnaire, along with the necessary explanations about the research objectives and informed consent, was sent to the students. To ensure responses, two reminder emails were also sent.

To ensure student participation in completing the questionnaires and prevent participant dropout, three reminder emails were sent to students. The introduction to the questionnaire included explanations about the study’s importance for educational planning. Students were requested to complete the questionnaire only once and avoid submitting duplicate responses.

### Data analysis

Since this study involved instrument development and construct validity assessment using a newly developed questionnaire, Exploratory Factor Analysis (EFA) was employed to identify the underlying factor structure. In addition, Confirmatory Factor Analysis (CFA) was conducted to validate the factor structure and assess model fit.

Descriptive statistics, including mean, standard deviation, and frequency, were also used. Data analysis was performed using SPSS version 24, with a confidence level of 95% and a significance level of 0.05.

### Ethics

This study was conducted in accordance with the ethical standards of the Research and Technology Deputy of Shiraz University of Medical Sciences. All participants received detailed information about the study objectives, procedures, and the voluntary nature of participation. Informed consent was obtained from all participants prior to completing the questionnaire. Participation was entirely voluntary, and respondents were assured of the anonymity and confidentiality of their responses. Furthermore, access to the completed questionnaire data was restricted and secured, accessible only through the primary investigator’s username and password. Participants retained the right to withdraw from the study at any time without any consequences.

## Results

### Demographic characteristics

Out of 350 distributed questionnaires, 298 were returned fully completed (response rate: ~85%). Among the participants, 145 were female (48.7%) and 153 were male (51.3%). Most students were aged 18–24 years (79.2%). The mean age was 22.71 years.

The largest proportion of students was from the Faculty of Medicine (*n* = 147, 49.3%), whereas the smallest proportion was from the Faculty of Paramedicine (*n* = 12, 4%).

Regarding academic level, 97 students (32.6%) were enrolled in BSc programs, while 201 (67.4%) were in professional doctoral programs.

Computer skills were self-reported as low by 85 students (28.5%), moderate by 157 (52.7%), and high by 56 (18.8%). All participants had access to at least one electronic device, and 47 students reported having access only to a mobile phone (Table 1).

**Table 1:** Demographic characteristics of participants.

Variables	Frequency	Percent (%)
Gender		
Female	145	48.7
Male	153	51.3
Age		
Age Group (18–24)	236	79.2
Age Group (≥ 25)	62	20.8
Average age = 22.71 ± 2.36		
Faculty		
Health	16	5.4
Nursing and Midwifery	32	10.7
Medicine	147	49.3
Paramedicine	15	5.0
Rehabilitation	12	4.0
Pharmacy	30	10.1
Dentistry	31	10.4
Nutrition	15	5.0
Educational Level		
BSc	97	32.6
Professional Doctorate	201	67.4
Computer Skills		
Low	85	28.5
Average	157	52.7
Proficient	56	18.8
Access to Electronic Devices		
Computer or Laptop + Mobile	251	84.2
Mobile Only	47	15.8
Total	298	100.0

### Main finding

Although the present study was conducted with a quantitative approach using factor analysis, the initial items were generated both through a review of previous research and theoretical foundations, as well as a brief qualitative study (Mini focus group).

#### Qualitative phase

To generate items for the questionnaire, a preliminary qualitative phase was carried out that combined a review of relevant literature with online focus group discussions.

A purposive sample of 15 faculty officials responsible for virtual education was selected, as they had direct experience with electronic and face-to-face learning processes.

Four focus group sessions were held via Adobe Connect between November 2021 to February 2022. Each session lasted approximately 120 min and was moderated by a researcher experienced in qualitative methods. In the first three sessions, participants were asked to brainstorm factors that influence learning in electronic and face-to-face settings, based on their observations of students and faculty, as well as their own professional experiences.

Contributions were shared verbally in online voice chat or in writing through the chat box. With participants’ consent, all sessions were recorded, reviewed, and coded by two researchers independently. In the initial stage, the items were developed based on a review of the relevant literature, existing questionnaires (e.g., CEQ), participants’ input from the focus group sessions, as well as the university conditions, infrastructure, and environments experienced by students in the Iranian context (including SUMS). This process resulted in an initial pool of 43 items. The list was subsequently returned to the participants for member checking. During the review process and qualitative analysis, it was identified that some items contained repetitive content, were similarly mentioned by multiple participants, or were less conceptually aligned with the main dimensions extracted from the discussions. Accordingly, duplicate, overlapping, or less relevant items were merged or removed. In the fourth focus group session, the remaining items were further discussed and refined. This iterative process led to a final set of 30 items, which were then carried forward to the quantitative phase.

#### Face validity

In the next phase, the face validity of the instrument was determined by three e-Learning specialists and 13 students, including those from Medicine^4^, Dentistry^1^, Nursing^2^, Health^2^, Health Management^1^, Nutrition^1^, and Pharmacy. The questionnaire was sent to them. Overall, most of them identified three questions as redundant and unnecessary. Additionally, four questions required grammatical corrections, and ultimately, 27 items were agreed upon.

#### Quantitative phase

In the quantitative phase, content validity was initially determined using the Content Validity Index (CVI) and Content Validity Ratio (CVR) from the perspective of 10 educational specialists (in fields such as Medicine, Medical Education, E-Learning, Laboratory Sciences, and Nursing Education).

#### Content validity

In the CVR method, the necessity of the items was assessed on a three-point Likert scale, and with 10 experts involved, a minimum agreement of approximately 0.625 was expected. For the CVI method, the questions were evaluated by specialists based on their relevance, clarity, and simplicity, with an anticipated minimum agreement of 79%.

#### Reliability

To determine the reliability of the questionnaire, it was administered to 298 students. After analysis, both the overall Cronbach’s alpha and item-specific (If item deleted) analyses were conducted. Ultimately, a total Cronbach’s alpha of 0.933 was obtained. Information regarding the determination of content validity and reliability is presented in Table 2.

**Table 2:** Descriptive characteristics of content validity and reliability of ELFEQ.

Items	Content validity				Reliability
	CVR		CVI		Cronbach’s Alpha
	Essential	Simple	Clear	Relevant	If Item Deleted
Q01	1.00	1.00	1.00	1.00	0.930
Q02	1.00	1.00	1.00	1.00	0.931
Q03	1.00	1.00	1.00	1.00	0.931
Q04	0.80	0.90	0.80	1.00	0.930
Q05	1.00	1.00	0.90	1.00	0.932
Q06	1.00	1.00	1.00	1.00	0.929
Q07	1.00	1.00	1.00	1.00	0.930
Q08	1.00	0.80	1.00	1.00	0.930
Q09	1.00	0.90	1.00	0.90	0.930
Q10	1.00	1.00	1.00	1.00	0.930
Q11	1.00	1.00	1.00	1.00	0.930
Q12	1.00	1.00	1.00	1.00	0.930
Q13	1.00	1.00	0.90	1.00	0.930
Q14	0.90	1.00	1.00	1.00	0.930
Q15	1.00	1.00	0.90	1.00	0.931
Q16	1.00	0.90	1.00	1.00	0.932
Q17	1.00	0.90	1.00	0.90	0.930
Q18	1.00	0.80	1.00	1.00	0.929
Q19	1.00	0.90	1.00	1.00	0.931
Q20	1.00	0.90	1.00	1.00	0.931
Q21	1.00	0.90	1.00	1.00	0.931
Q22	1.00	1.00	1.00	1.00	0.930
Q23	1.00	1.00	0.90	1.00	0.932
Q24	1.00	1.00	1.00	1.00	0.929
Q25	1.00	1.00	1.00	1.00	0.928
Q26	1.00	1.00	1.00	1.00	0.930
Q27	1.00	1.00	1.00	1.00	0.930
Total	**0.989**	**0.959**	**0.978**	**0.993**	0.930
		CVI _Total_ = 0.976			0.933

Significant values are in bold.

#### Construct validity

In this study, construct validity was assessed using EFA and CFA. After extracting the initial items based on the literature review and focus group discussions, the questionnaire—comprising 27 items measured on a 5-point Likert scale—was administered to 350 medical students at SUMS, of which 298 completed questionnaires were included in the analysis. The results were analyzed using exploratory factor analysis.

**Fig. 1:**
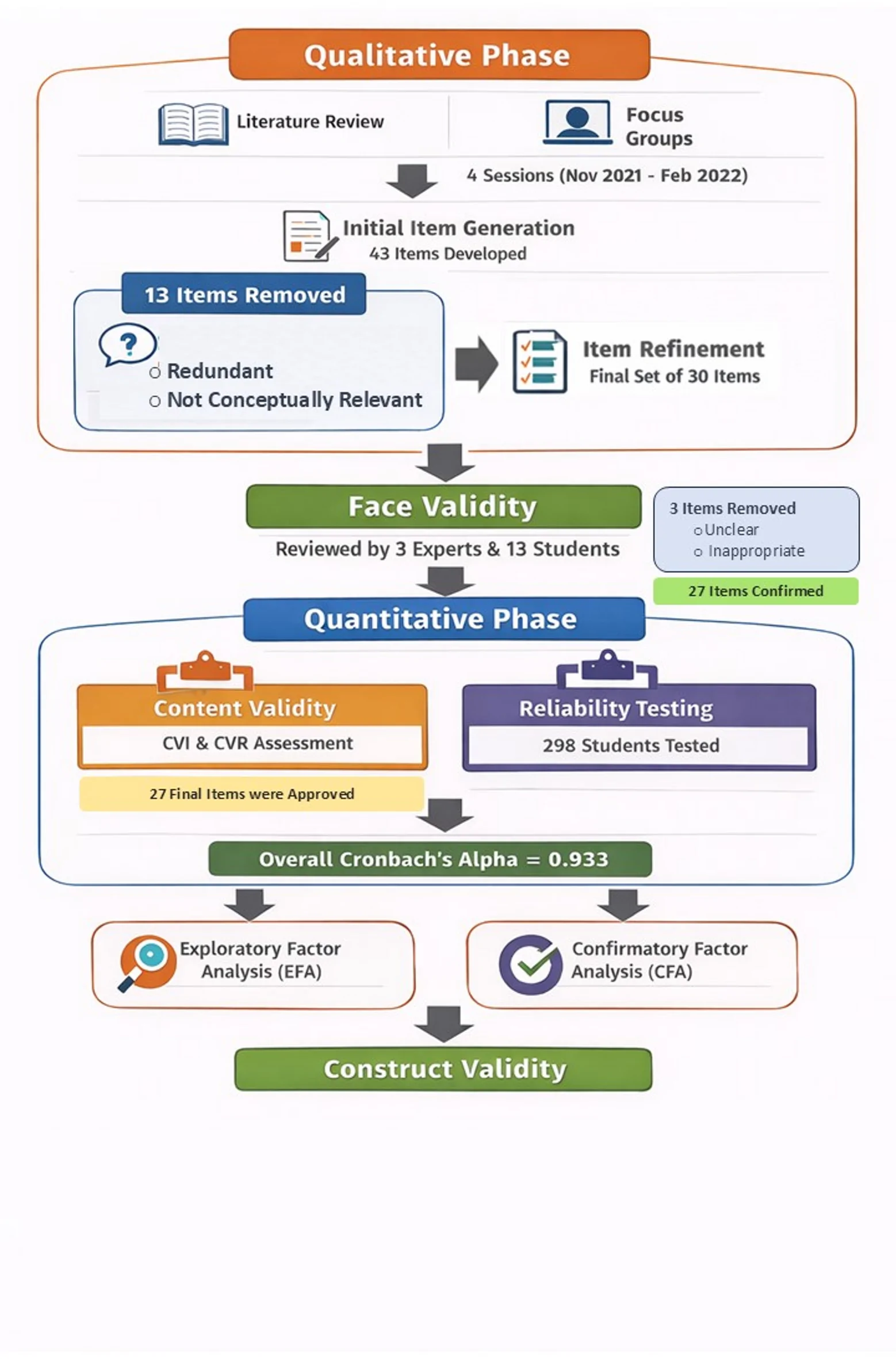
Stages of the questionnaire development and validation process.

The overall outline of the research stages is shown in Fig. 1.

### Exploratory factor analysis

In EFA, specific criteria must be evaluated both prior to and following the analysis:

#### Criteria before conducting EFA

To ensure that the sample size is adequate, the Kaiser-Meyer-Olkin (KMO) Measure of Sampling Adequacy and Bartlett’s Test of Sphericity were used. The results of these tests are presented in Table 3. According to Table 3, the KMO value of 0.920 and P-value < 0.001 indicate the suitability of the test and the adequacy of the sample size.

**Table 3:** KMO and Bartlett’s Test of factor analyzing the ELFEQ.

Kaiser-Meyer-Olkin Measure of Sampling Adequacy	0.920
Bartlett’s Test of Sphericity	
Approx. Chi-Square	4433.833
df	351
Sig.	< 0.001

#### Criteria after conducting EFA

##### Total Variance Explained and Scree Plot

**Fig. 2:**
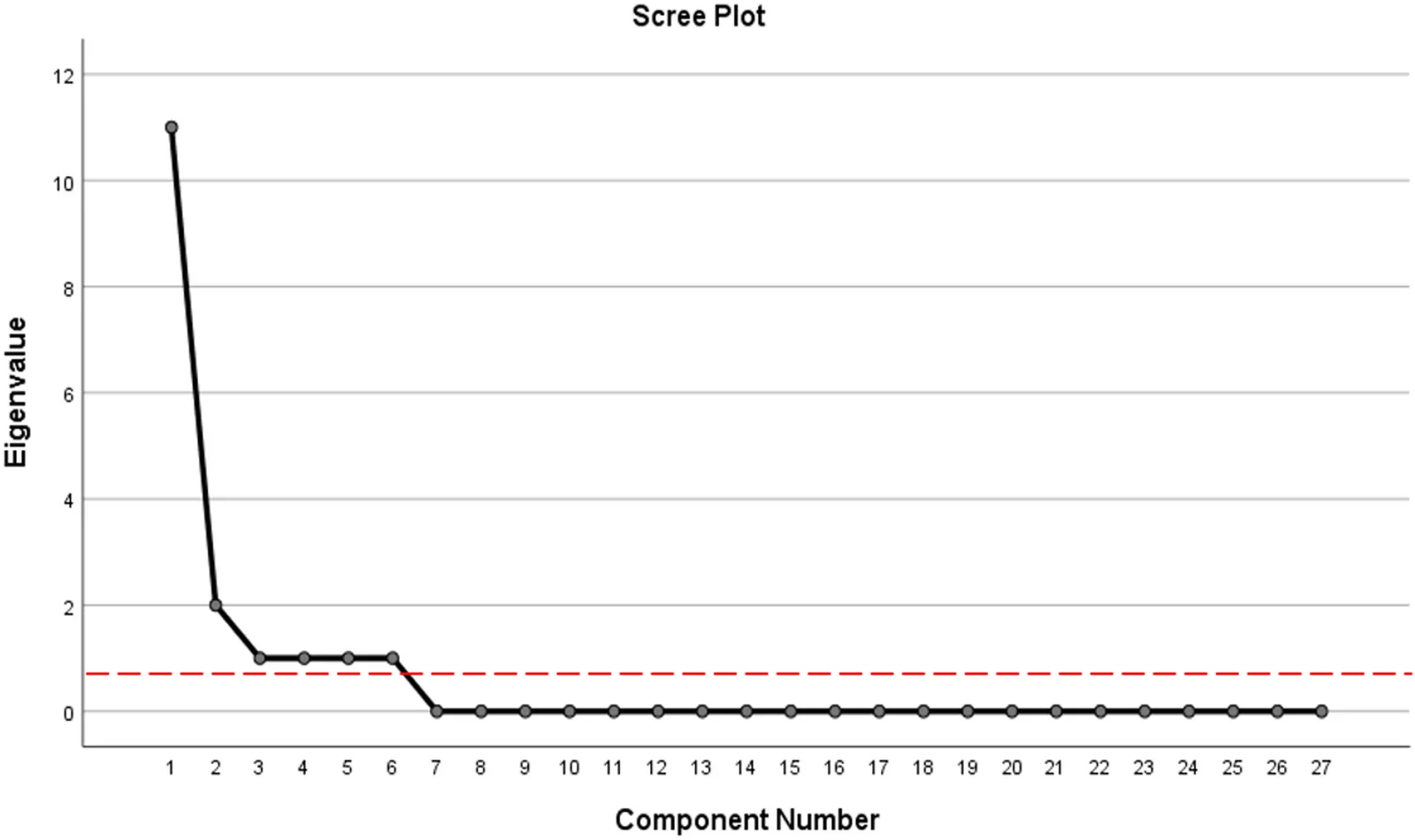
Scree plot of exploratory factor analysis.

Based on Table 4, the number of factors derived from the EFA is shown. According to Table 4, the Initial Eigenvalues indicate that the six factors extracted from the factor analysis account for approximately 70.523% of the variance in the construct of the learning environment, suggesting that this tool has a strong fit. The first factor exhibits the highest loading at %42.506, while the subsequent factor loadings are shown in Table 4. In addition, Fig. 1 provides a visual representation of the factor analysis, including the scree plot (Fig. 2).

**Table 4:** Total variance explained of EFA of ELFEQ.

Component	Initial Eigenvalues			Extraction sums of squared loadings			Rotation sums of squared loadings		
	Total	% of Variance	Cumulative %	Total	% of Variance	Cumulative %	Total	% of Variance	Cumulative %
1	11.477	42.506	42.506	11.477	42.506	42.506	5.240	19.406	19.406
2	2.450	9.075	51.582	2.450	9.075	51.582	3.431	12.708	32.115
3	1.653	6.122	57.704	1.653	6.122	57.704	3.025	11.202	43.317
4	1.304	4.829	62.533	1.304	4.829	62.533	2.960	10.961	54.278
5	1.149	4.257	66.790	1.149	4.257	66.790	2.302	8.524	62.803
6	1.008	3.734	**70.523**	1.008	3.734	70.523	2.085	7.721	70.523
7	0.842	3.120	73.643						

Extraction method: exploratory factor analysis.

Significant values are in bold.

#### Communalities

Communalities range from 0 to 1, with values closer to 1 indicating that a large portion of a variable’s variance is explained by the extracted factors. Values below 0.40 suggest weak correlations or possible unidentified factors. As shown in Table 5, all communalities range from 0.406 to 0.786, exceeding the expected threshold of 0.40. This indicates that all items demonstrate acceptable to very good levels of shared variance, with Q9, Q16, and Q08 showing the highest values related to class interaction, and Q14 and Q15 presenting the lowest but still acceptable values. (Table 5).

**Table 5:** Communalities of each item of ELFEQ.

Items	Communalities
Q01. Scientific content of the courses was useful and informative	0.693
Q02 Educational content was well-designed based on audio visually.	0.717
Q03. The volume of course content provided during the semester was appropriate.	0.541
Q04. I was able to access the course content and review/practice it at my own pace.	0.645
Q05. Assignments and exercises were effective in enhancing my learning.	0.602
Q06. Assignments and learning activities encouraged me to study the course content.	0.604
Q07. Discussions and conversations during the course were informative.	0.680
Q08. Discussions encouraged greater participation and engagement among students.	0.754
Q09. Discussions encouraged me to reflect on my own and my classmates’ opinions.	0.786
Q10. Discussions helped me learn from my classmates.	0.739
Q11. Quizzes and exams were effective in reviewing and learning the course material.	0.744
Q12. Quizzes an Being comfortable and motivated me to study the course.	0.744
Q13. Quizzes and exams helped me pay more attention to key points in the lessons.	0.665
Q14. The time allocated for answering quizzes was sufficient.	*0.406*
Q15. I completed the quizzes and exams myself without help from classmates.	*0.446*
Q16. There were good interaction and question–answer exchange in the class.	0.777
Q17. I felt that my classmates played an effective role in my learning.	0.538
Q18. I could ask my questions freely in class without fear of judgment.	0.564
Q19. When the instructor asked questions, I could respond more comfortably.	0.735
Q20. During lessons, I was focused and less distracted by my surroundings.	0.543
Q21. Seeing the instructor synchronous interaction enhanced my learning.	0.553
Q22. Attending class sessions was important to me.	*0.494*
Q23. Courses were presented by instructors in an organized and sequential manner.	0.648
Q24. There was adequate teacher-students interaction throughout the course.	0.695
Q25. There were opportunities for student participation during the course.	0.506
Q26. Instructors motivated students to study and learn.	0.589
Q27. I benefited from the instructors’ ethical behavior and role modeling.	0.558

Expected value of Communalities > 0.40.

.

#### Varimax rotation analysis

Using exploratory factor analysis and Varimax rotation, six factors were initially extracted. However, after examining the correlations among items and the number of items associated with each factor, a total of six factors were ultimately identified. The final factor loadings and the number of items per factor in the questionnaire are presented in Table 6. From the psychometric analysis and the extraction of concepts as *“E-Learning and Face-to-Face Learning experience Questionnaires”* (ELFEQ), the most significant factors were categorized as follows: Peer assisted learning (6 items), Teacher-student interaction (7 items), Examination (5 items), Being comfortable (3 items), Content (3 items), and Assignments (3 items). (Table 6, and Appendix 1)

**Table 6:** Rotated factor matrix of ELFEQ.

No	Factor 1	Factor 2	Factor 3	Factor 4	Factor 5	Factor 6	Reliability (α)
							For each factor
Q7	**0.663**	0.245	0.214	0.096	0.154	0.342	α = 0.865
Q8	**0.812**	0.188	0.173	0.074	0.074	0.197	
Q9	**0.825**	0.143	0.212	0.116	0.197	0.108	
Q10	**0.784**	0.233	0.277	0.128	0.155	0.065	
Q16	**0.772**	0.252	0.242	0.114	0.082	0.067	
Q17	**0.770**	0.227	0.189	0.079	0.139	0.072	
Q21	0.387	**0.606**	0.105	0.373	0.064	0.157	α = 0.858
Q22	0.043	**0.479**	0.054	0.450	0.108	0.235	
Q23	0.252	**0.722**	0.255	0.109	0.119	0.033	
Q24	0.491	**0.665**	0.153	0.105	0.094	0.125	
Q25	0.497	**0.599**	0.153	0.184	0.122	− 0.101	
Q26	0.275	**0.514**	0.333	0.214	0.232	0.048	
Q27	0.157	**0.661**	0.231	0.095	0.193	0.197	
Q11	0.459	0.211	**0.635**	0.081	0.148	0.098	α = 0.825
Q12	0.431	0.244	**0.664**	0.076	0.169	0.193	
Q13	0.328	0.168	**0.702**	0.148	0.187	0.158	
Q14	0.164	0.162	**0.741**	0.181	0.187	0.055	
Q15	0.203	0.381	**0.528**	0.021	0.056	0.232	
Q18	0.174	0.009	0.156	**0.877**	0.074	0.030	α = 0.813
Q19	0.136	0.160	0.062	**0.882**	0.080	0.097	
Q20	0.078	0.308	0.178	**0.706**	0.033	0.301	
Q1	0.124	0.157	0.230	0.107	**0.732**	0.015	α = 0.775
Q2	0.222	0.165	0.156	− 0.059	**0.789**	0.004	
Q3	0.182	0.080	0.125	0.135	**0.700**	0.270	
Q4	− 0.035	0.094	− 0.064	0.369	0.481	**0.517**	α = 0.763
Q5	0.212	0.118	0.171	0.250	0.137	**0.794**	
Q6	0.287	0.135	0.301	0.100	0.063	**0.745**	

Eagen Value (Rotation Sums of Squared Loadings)	70.523						
The number of initial factors after EFA	Six Factor						
Factor 1: Peer Assisted Learning (α = 0.865) Factor 2: Teacher-Student Interaction (α = 0.858) Factor 3: Examination and Quizzes (α = 0.825)	Factor 4: Being comfortable (α = 0.813) Factor 5: Content (α = 0.775) Factor 6: Assignments (α = 0.763)						

Significant values are in bold.

Based on Table 6, each factor includes the following items:

- Factor 1 – Peer-Assisted Learning: Q7, Q8, Q9, Q10, Q16, Q17.
- Factor 2 – Teacher–Student Interaction: Q21, Q22, Q23, Q24, Q25, Q26, Q27.
- Factor 3 – Examinations and Quizzes: Q11, Q12, Q13, Q14, Q15.
- Factor 4 – Being Comfortable: Q18, Q19, Q20.
- Factor 5 – Content: Q1, Q2, Q3,
- Factor 6 – Assignments: Q4, Q5, Q6.

The exploratory factor analysis. identified six main factors explaining 70.52% of the total variance, all demonstrating satisfactory to excellent internal consistency. Cronbach’s alpha values for the six factors ranged from 0.763 to 0.865, indicating strong reliability across dimensions. After conducting EFA and extracting factors and items, the final questionnaire was designed, which can be seen in Appendix 1.

### Confirmatory factor analysis

**Fig. 3:**
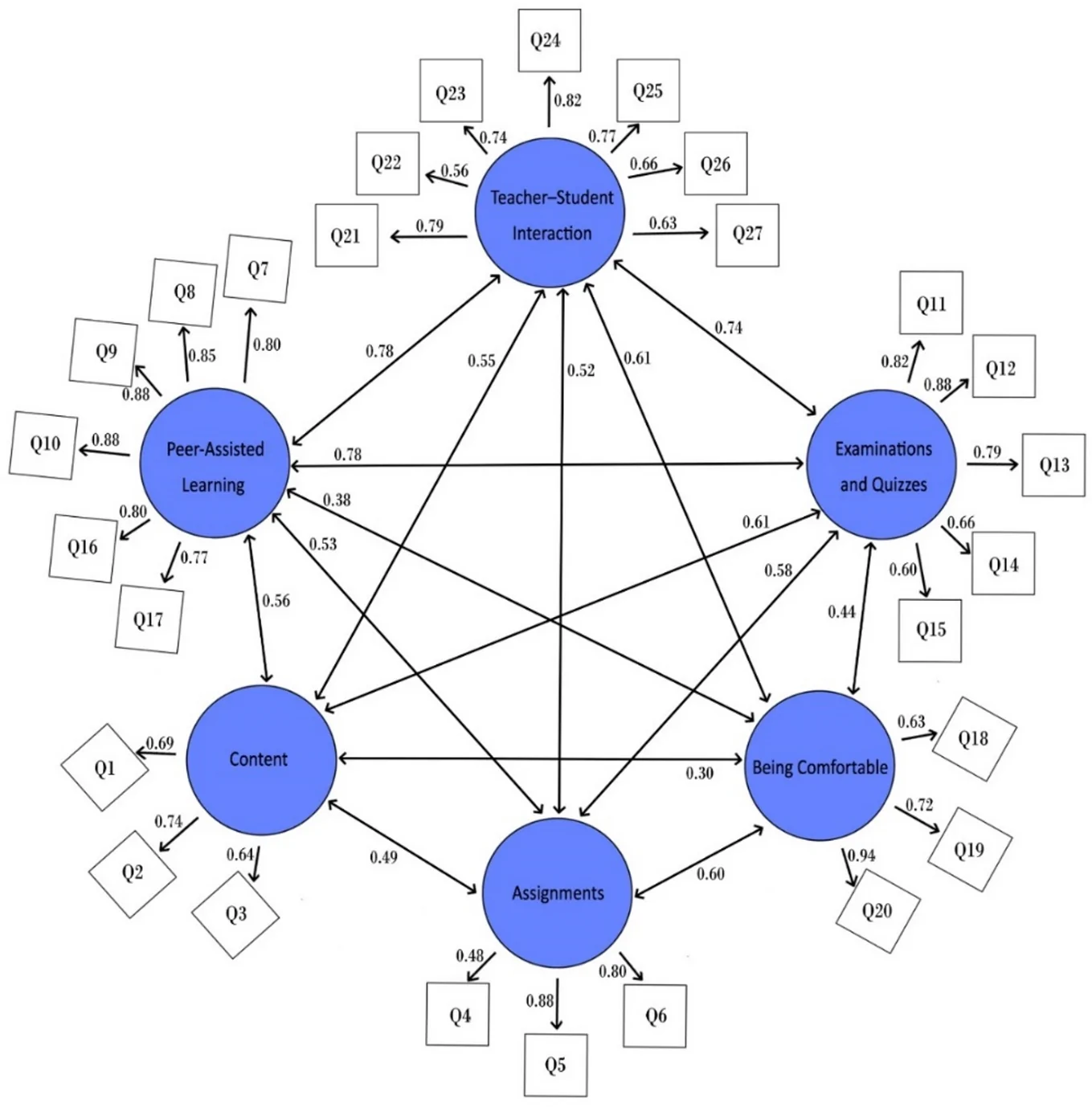
Confirmatory factor analysis of the six-factor structure of the ELFEQ.

Following the EFA, a confirmatory factor analysis was conducted to validate the six-factor structure of the ELFEQ, as illustrated in Fig. 3.

A six-factor confirmatory factor analysis (CFA) was conducted to evaluate the construct validity of the ELFEQ. While the initial model demonstrated an acceptable but suboptimal fit, four theoretically defensible correlated error terms (Q18–Q19, Q16–Q17, Q26–Q27, and Q3–Q4) were added based on modification indices. The revised model showed substantially improved fit indices. Overall, the revised six-factor measurement model provides strong evidence for the construct validity of the ELFEQ (Table 7).

**Table 7:** Fit indices for the revised CFA model of the ELFEQ.

Index	Modified model value	Acceptable range	Interpretation
χ²/df	668.039/305 = 2.19	Less than 3 = Good	Acceptable
CFI	0.929	Above 0.90 Good; Above 0.95 Excellent	Good
TLI	0.919	Above 0.90 Good; Near 0.95 Excellent	Good
RMSEA	0.063	0.05–0.08 = Good; <0.05 = Excellent	Good
SRMR	0.059	Less than 0.08 Good; <0.05 = Excellent	Good

The revised model showed substantially improved fit indices: CFI = 0.929, TLI = 0.919, RMSEA = 0.063, and SRMR = 0.059. According to commonly recommended cutoffs, the CFI and TLI values indicate good model fit, and the RMSEA and SRMR fall within the acceptable range. Consequently, the revised six-factor ELFEQ model exhibits an adequate fit, and its factor structure shows strong consistency with the sample data.

## Discussion

This study aimed to develop and validate a questionnaire designed to evaluate key factors influencing students’ learning experiences in both face-to-face (F2F) and e-learning environments. The psychometric properties of the instrument, including reliability, content validity, and construct validity, were rigorously assessed. Furthermore, factor loadings were used to determine the relative importance of each component. The results of these analyses are presented in the following sections.

Overall, the findings indicate that students’ learning experiences in both modalities are multidimensional and cannot be adequately captured by a single cognitive or performance-based indicator, highlighting the complexity of contemporary educational environments.

### Psychometric characteristics of instrument

The results of the research indicated that the ELFEQ tool has a good fit for assessing learning in both face-to-face and electronic environments. The findings can be discussed in two parts: first, the psychometric characteristics of the tool, and second, the priorities and significance of the identified components.

#### Reliability

Reliability is a critical characteristic of any measurement tool, as it indicates how consistently the tool measures a construct across different contexts and over times^32^. In this study, the reliability of the ELFEQ tool was assessed using internal consistency, measured by Cronbach’s alpha. According to DeVellis^33^, an instrument with a Cronbach’s alpha of 0.70 or higher is generally considered reliable. The obtained reliability coefficient of 0.933 demonstrates that the ELFEQ tool has an excellent level of internal consistency. This high value, which is close to 1, confirms that the instrument provides stable and consistent results, ensuring that the data collected are trustworthy^34^. The very high reliability coefficient further indicates that the instrument provides stable measurement across items, suggesting that the constructs are internally coherent and conceptually well-defined.

In this study, the reliability of each item in the ELFEQ tool was also examined under the condition of “if item deleted.” This method involved removing each item one at a time from the reliability analysis to assess the internal consistency of the remaining items. When an item is excluded from the analysis, the resulting Cronbach’s alpha value is recalculated. If the overall reliability increases upon removing an item, it suggests that the item has low internal consistency with the overall scale and may need revision. Conversely, if the overall reliability decreases when an item is removed, it indicates that the item contributes positively to the internal consistency of the instrument. The results of this analysis showed that removing any individual item led to a decrease in the overall reliability coefficient. This outcome confirms that each item in the ELFEQ tool maintains a strong internal consistency with the overall scale, indicating that all items are appropriate and contribute meaningfully to measuring the intended constructs.

#### Validity

Validity encompasses several dimensions, including face validity, content validity, and construct validity. Content validity assesses whether the instrument adequately covers the construct it intends to measure^35^. In the present study, content validity was assessed using two methods: Content Validity Index (CVI) and Content Validity Ratio (CVR). The results indicated that both indices were confirmed with values greater than 0.90. According to Lawshe (1975), the acceptable CVR value depends on the number of experts participating in the validity assessment; for a sample size of 10 experts, a CVR of approximately 0.625 is considered acceptable^36^. For the CVI, three criteria—relevance, simplicity, and clarity of the items—were evaluated and confirmed to be at an excellent level. Waltz and Bausell (1981) suggest that a minimum CVI of 0.79 is acceptable for each item and for the overall scale^37^. They also state that if the CVI is around 0.70, those items should be revised. Given the results obtained in this study, it appears that the ELFEQ tool possesses adequate content validity. High content validity indicates that the questionnaire is appropriate for capturing the intended data, enhancing the overall quality and reliability of research findings.

##### Construct validity

This criterion evaluates whether the instrument truly measures the theoretical construct it claims to measure, often through factor analysis or correlations with other established measures. Based on the results, the KMO value reported is 0.920. The KMO statistic ranges from 0 to 1, with higher values indicating that the data is suitable for factor analysis. A KMO value above 0.90 is considered “meritorious,” suggesting excellent sampling adequacy^38^. This high KMO value indicates that correlations between items are sufficiently strong to perform factor analysis, meaning that the dataset is appropriate for identifying underlying factors.

Additionally, Bartlett’s Test of Sphericity tests^39^ whether the correlation matrix is an identity matrix, which would indicate that variables are unrelated and unsuitable for structure detection. A significant result (*p* < 0.05) indicates significant correlations among variables, justifying the use of factor analysis. In this study, the very low p-value (< 0.001) suggests that the null hypothesis can be rejected, confirming significant relationships among items in the dataset.

Exploratory factor analysis, combined with variance results from the exploratory factor analysis of the ELFEQ, revealed that the cumulative percentage of variance explained reaches 70.523% after the sixth component. Educationally, this factor structure demonstrates that students’ experiences in both face-to-face and online environments are organized around core dimensions of interaction, cognitive engagement, assessment practices, and psychological comfort. This finding underscores that learning experiences are multidimensional and context-sensitive rather than unidimensional.

To determine which variable corresponds to each factor, a specific cutoff for loading values is established, which may differ depending on the study context^40^. For biological markers, this cutoff is generally set around |0.5^41^, while in nutritional studies, it tends to be approximately |0.3^42^. In other research types, this threshold may vary depending on correlations among independent variables^43^.

Following the exploratory factor analysis (EFA), a six-factor confirmatory factor analysis (CFA) was conducted to further examine the construct validity of the ELFEQ. The initial model demonstrated an acceptable but suboptimal fit; therefore, four theoretically justifiable correlated error terms (Q18–Q19, Q16–Q17, Q26–Q27, and Q3–Q4) were added based on modification indices. The revised model showed improved fit indices.

The results indicated χ²/df = 2.19, Comparative Fit Index (CFI) = 0.929, Tucker–Lewis Index (TLI) = 0.919, Root Mean Square Error of Approximation (RMSEA) = 0.063, and Standardized Root Mean Square Residual (SRMR) = 0.059. Based on commonly recommended criteria, these values fall within acceptable ranges, suggesting an adequate model fit^44–46^. Overall, in line with the findings from the EFA, the CFA results indicate that the six-factor structure of the ELFEQ demonstrates an acceptable level of construct validity. While preliminary evidence supports the ELFEQ ’s utility in Iranian medical contexts, explaining 70% of variance, the remaining 30% remains unexplained. This may be influenced by factors not captured in the questionnaire, such as students’ access to technology, prior e-learning experience, or individual learning preferences. Therefore, while the ELFEQ shows promise, its applicability should be interpreted cautiously, and further research is needed to explore additional factors affecting learning experiences.

Beyond statistical validation, examining factor loadings provides insight into students’ priorities and highlights which dimensions are most central to their perceived learning experiences during the pandemic.

### Importance of the components of the ELFEQ

In addition to the psychometric dimensions of this study, the factor loadings of the extracted components indicate the importance and priority of these factors from the perspective of students during the COVID-19 pandemic. Based on the findings, six distinct factors were identified: Peer Assisted Learning, Teacher–Student Interaction, Examination and Quizzes, Comfort, Content, and Assignments. These factors provide a nuanced understanding of the elements contributing to students’ learning experiences.

An important point regarding alignment with existing theories is that the two highest-ranked factors from students’ perspectives—Peer Assisted Learning and Teacher–Student Interaction—both emphasize the importance of interaction and communication with instructors and peers. These findings are strongly aligned with the Community of Inquiry (CoI) model^47^, which highlights teaching presence, social presence, and cognitive presence as essential elements of meaningful learning experiences. In this study, Teacher–Student Interaction corresponds to teaching presence, while Peer Assisted Learning and Comfort reflect social presence. Content, Assignments, and Examinations are conceptually consistent with cognitive presence, underscoring the role of structured learning activities in knowledge construction.

Additionally, the results can be interpreted through Transactional Distance Theory^48^, which posits that the quality of interaction and the structure of the learning environment determine the psychological and communicative distance between learners and instructors. The prominence of interaction-related factors in this study supports the theory’s assertion that reducing transactional distance enhances learning experiences, particularly in online environments. From a constructivist perspective^49^, learning is socially mediated and developed through interaction with peers and instructors. The strong factor loadings of interaction-related dimensions reinforce the importance of collaborative learning environments in both face-to-face and electronic modalities.

#### Peer assisted learning

The significance of Peer-Assisted Learning highlights the importance of collaborative learning environments, supporting prior research on the role of peer interactions in improving outcomes^49,50^. Providing opportunities for students to engage with each other—both in e-learning and face-to-face (F2F) settings—enhances comprehension and retention. Educational institutions may benefit from structured peer learning programs, especially in online contexts where social interactions are less spontaneous.

In medical education, which relies on interdisciplinary teamwork, peer learning is a key contributor to knowledge and skill development. Learning from peers strengthens understanding and practical competencies.

These findings align with Li et al. (2024), who reported that F2F students outperformed online peers in attendance, group dynamics, and overall proficiency, highlighting the continued value of traditional classroom approaches in fostering interaction and essential academic and professional skills^20^.

#### Teacher–student interaction

The factor of Teacher–Student Interaction underscores the critical role of effective communication in the learning process. Research indicates that positive interactions between educators and students can significantly enhance student motivation and engagement^51^. In e-learning contexts, where direct interaction may be limited, educators may employ strategies such as regular feedback, virtual office hours, and interactive discussion forums to maintain effective communication with students. Firdousi et al. (2024) examined the impact of different teaching modalities—traditional, online, and hybrid—on student academic performance and satisfaction^52^. The findings suggest that perceived instructional quality plays a key role in student satisfaction across all learning modes. Students reported higher satisfaction when their learning needs were addressed through effective pedagogical strategies, including interactive activities and timely feedback. Overall, these findings highlight the importance of integrating diverse teaching approaches in curriculum design to accommodate different learning needs and enhance the overall educational experience.

#### Examination and quizzes

The recognition of Examination and Quizzes as a distinct factor highlights the importance of assessment methods in influencing students’ learning experiences. Effective assessments serve not only to measure knowledge but also to encourage students to actively engage with course material^53^. Educational institutions should ensure that their assessment methods align with learning objectives and consider integrating formative assessments that offer continuous feedback, rather than relying exclusively on high-stakes testing.

#### Being comfortable

The aspect of Being Comfortable emphasizes how students’ emotional and psychological states affect their learning experiences. Comfort in both physical and virtual learning environments can significantly influence engagement and overall satisfaction^54^. Educators should aim to create inclusive and supportive environments that cater to students’ needs, whether through flexible learning options or by fostering a sense of community among learners. This factor can inherently relate to various other aspects, including the previously mentioned factors. In the research conducted by Hollister et al. (2022), many students reported difficulties in maintaining connections with their peers and instructors, as well as challenges in managing the pace of their coursework^12^.

#### Content

Content quality has emerged as a vital factor influencing student learning experiences. This highlights the necessity for educational materials to be relevant, engaging, and accessible across different learning modalities. Institutions should prioritize curriculum development that integrates diverse teaching methods and resources to accommodate varying learning needs.

Si (2022) reports a direct relationship between higher content quality and increased student satisfaction in e-learning environments^55^. The study identifies key determinants of content quality, including sufficiency, conciseness, instructional design, and relevance to learners’ needs. It also suggests that effective learner–content interaction, in which students actively engage with materials, can enhance the overall educational experience.

Similarly, Mohammad (2023) examines how online learning environments influence student satisfaction and engagement^56^. The study emphasizes the importance of creating an interactive learning environment that encourages participation and supports student engagement. It further highlights that access to high-quality resources and ease of use are critical for positive learning experiences.

Overall, these findings suggest that when educational materials are relevant and well-designed, students are more likely to achieve their learning objectives, supporting the need for diversified instructional approaches. In addition, students reported several limitations of electronic learning environments, including technical issues such as power outages, internet instability, lack of private study space, and administrative challenges. However, they also perceived online learning as safer in terms of physical accessibility and convenience. Moreover, students viewed the integration of online and face-to-face learning as beneficial, as it may reduce costs and travel time while providing access to independent learning resources^56^.

#### Assignments

The factor related to Assignments highlights the significance of well-structured tasks in fostering active learning. Assignments should be crafted not only to assess knowledge but also to stimulate critical thinking and the application of learned concepts^57^. Providing clear guidelines and timely feedback can enhance student engagement and motivation. Notably, this component ranked last among the factors extracted from EFA and exhibited the lowest factor loading. This may be attributed to the advantages of completing assignments in a F2F environment, such as being physically present in class, receiving direct feedback from instructors^56^, and learning collaboratively with peers^49^. However, online environments offer flexibility that can help overcome spatial and temporal limitations.

In a broader overview of the six factors derived from exploratory factor analysis, which serve as a basis for comparing students’ learning experiences in F2F and electronic environments, several noteworthy points emerge. This research was conducted during a period when students had access exclusively to online environments. The components with the highest factor loadings reflect the primary concerns of students in electronic settings, which they perceived as distinct from those in F2F contexts. The first two factors pertain to “Interaction,” indicating that students view interaction as a crucial element of education that requires greater attention. The third factor relates to formative and summative electronic assessments, which showed significant differences in implementation between electronic and F2F environments and became a major concern for both instructors and students during the pandemic. Students faced challenges such as unequal access to electronic devices and concerns about course completion due to network instability and technical issues.

Conversely, the remaining factors—comfort, content access, and assignment completion—showed lower factor loadings. Despite the challenges of electronic environments, learning management systems provided better opportunities for solving exercises and accessing educational content compared to traditional face-to-face settings. Moreover, due to limited resources, instructors adapted by developing digital learning materials rather than relying solely on PowerPoint presentations.

Overall, both F2F and electronic environments, each with their own advantages, can support student learning through a blended approach. Although the lack of face-to-face interaction during the pandemic negatively affected interpersonal connections—particularly among undergraduate students—this limitation has been reduced in the post-pandemic period. Consequently, educators and students can now leverage the strengths of both modalities not out of necessity or crisis, but through intentional selection of appropriate blended learning strategies.

It should be noted that the focus on both face-to-face and electronic learning environments has attracted considerable attention in universities worldwide. While the effectiveness of these modalities may be influenced by contextual factors, numerous studies have identified similarities in student experiences and perceptions across different settings.

Our findings regarding the psychometric robustness of the ELFEQ tool and the identified factors align with international research. For example, Hilton et al. (2020) reported that the effectiveness of learning environments cannot be attributed solely to delivery mode (online vs. face-to-face). They emphasized that instructor variability in teaching style, as well as student characteristics such as age and prior experience with online platforms, may influence learning outcomes^25^.

Koja and Abazaj (2024) highlighted the importance of internet infrastructure as a key determinant of online learning effectiveness. They also noted that students’ preferences for online platforms are shaped by individual personality traits. Their findings further suggest that content quality plays a central role, as students consider well-designed and engaging materials essential for meaningful learning experiences^23^.

Similarly, Mather and Sarkans (2018) identified multiple determinants of effective learning environments, including peer and instructor interaction, assignment design, learning materials, feedback, and group collaboration^26^.

In line with the present study, Siddiqui et al. (2024) reported advantages of online learning such as flexible access to resources, lecture recording, and reduced commuting time, alongside disadvantages including limited interaction, self-regulation challenges, and social isolation^18^.

Sun (2023) compared online and face-to-face learning using the Course Experience Questionnaire (CEQ) and the Online Course Experience Questionnaire (OCEQ), reporting differences in student satisfaction, teaching effectiveness, clarity of goals, and assessment quality^24,58,59^. These differences were partly attributed to reduced social interaction in online environments, consistent with constructivist theory.

Overall, these studies suggest that while both modalities offer distinct advantages, learning experiences are strongly shaped by interaction quality, instructional design, and contextual factors rather than delivery mode alone.

It should be noted that instruments such as the CEQ and its online adaptations have been widely used to evaluate the quality of educational courses. However, to clarify the conceptual positioning of the present instrument, it is important to compare it with the CEQ and its online versions. The CEQ is a well-established instrument in higher education for assessing students’ perceptions of course quality, primarily measuring dimensions such as good teaching, clear goals and standards, appropriate assessment, workload, and generic skill development^58^.

Although there are conceptual overlaps between the CEQ and the present instrument, particularly in areas such as teacher–student interaction and assessment, important differences exist. The primary objective of this study was not to evaluate course quality or teaching effectiveness per se, but to examine students’ learning experiences across face-to-face and online environments during the COVID-19 pandemic.

During this period, the abrupt transition between learning modalities led to changes in interaction patterns, peer engagement, psychological comfort, and classroom dynamics. In this context, dimensions such as peer-assisted learning, psychological comfort, and instructor-related motivational factors emerged as distinct constructs not explicitly operationalized within the traditional CEQ framework.

Moreover, although some domains overlap with the CEQ, the factor structure identified in this study reflects a different organization of learning experience components within this context. Therefore, the present instrument should be considered a context-sensitive tool rather than a replacement for the CEQ, designed to capture learning experiences in transformed educational environments, particularly in the post-pandemic era.

International evidence supports the relevance of these dimensions across educational contexts, suggesting that interaction, content quality, assessment, and comfort consistently influence students’ learning experiences. However, differences in infrastructure, cultural attitudes toward technology, and pedagogical practices may affect the relative importance of these factors, indicating the need for further cross-cultural validation.

Blended learning in Iran is shaped by contextual and cultural factors. In collectivist cultures such as Iran, social interaction plays an important role in learning, which may influence the acceptance and effectiveness of blended learning approaches. Evidence suggests that students in Middle Eastern countries generally prefer a combination of online and face-to-face instruction, although this preference depends on institutional support and infrastructure availability^60^. Furthermore, during the COVID-19 pandemic, medical education programs at institutions such as Tehran University of Medical Sciences adopted blended learning models to support clinical training in resource-constrained settings^61^.

Overall, the integration of psychometric evidence, theoretical alignment, and empirical comparisons suggests that the ELFEQ provides a useful framework for understanding students’ learning experiences in both modalities. However, the use of a single-university sample limits generalizability. Therefore, future studies in diverse cultural and institutional contexts, as well as cross-national validation, are recommended to strengthen external validity and further explore contextual influences on learning experiences.

## Conclusion

In summary, the exploration of students’ learning experiences in both face-to-face and electronic environments reveals significant insights into the evolving landscape of education. The findings highlight that interaction remains a cornerstone of effective learning, emphasizing the importance of engagement between students and instructors, as well as among peers. The challenges posed by the pandemic, such as unequal access to technology and concerns over assessment integrity, have underscored the need for adaptive strategies that prioritize student needs. Moreover, while electronic environments offer flexibility and increased access to resources, they also necessitate a thoughtful integration of pedagogical practices that can enhance learning outcomes. The development of rich electronic content and innovative assessment methods has the potential to bridge gaps created by physical distancing. As educational institutions move forward, embracing a blended learning model that combines the strengths of both face-to-face and online modalities can foster a more inclusive and effective educational experience. By leveraging technology while maintaining essential human interactions, educators can create an enriching learning environment that not only meets the diverse needs of students but also prepares them for a dynamic future. Ultimately, this approach can lead to improved academic performance and greater student satisfaction, ensuring that education remains resilient and responsive in an ever-changing world.

## Limitations and future research directions

This study has several limitations. First, the sample was drawn from a single Iranian medical university and included only undergraduate and professional doctorate students. Consequently, the findings may not be generalizable to other institutions, disciplines, educational levels, or cultural contexts. The relatively homogeneous cultural background of participants, including collectivist norms in Persian education, may also have influenced peer interactions and learning experiences. Future research is recommended to replicate the validation process in multi-center studies with more diverse populations, including postgraduate students, to enhance the external validity of the instrument.

Second, this study focused solely on student perspectives. Faculty viewpoints and other educational stakeholders were not included, which could provide complementary insights into the learning process. Future studies are encouraged to incorporate multiple perspectives to achieve a more comprehensive understanding of educational experiences.

Third, the study employed a cross-sectional design, capturing student experiences at a single point in time (2021–2022). As a result, it does not account for potential changes in learning experiences over time as educational practices evolve.

Furthermore, since the aim of this study was to compare factors influencing students’ learning experiences in both e-learning and face-to-face environments, only undergraduate students were included. This decision was made because, at Shiraz University of Medical Sciences (SUMS), the structure, content, and management of postgraduate courses differ substantially from those of undergraduate programs.

Finally, although both exploratory factor analysis (EFA) and confirmatory factor analysis (CFA) were conducted to examine the factor structure and construct validity of the instrument, further validation is still warranted. Future research should assess additional psychometric properties, such as measurement invariance, differential item functioning, and test–retest reliability, and perform cross-validation using independent samples in diverse populations to confirm the stability and generalizability of the findings.

## Future research and educational implications

Future studies should replicate this research across multiple disciplines and cultural contexts to validate the identified factors and explore how cultural norms, such as collectivism in Persian education, influence learning experiences. Including faculty perspectives could provide a more comprehensive understanding of teaching and learning dynamics in online and face-to-face environments. Longitudinal studies may also help capture how these factors evolve over time, particularly as educational practices continue to adapt post-pandemic. In addition, future research should examine the relationship between students’ perceived emotional experiences and objective educational performance indicators, as well as track changes in emotional and psychological dimensions over time.

From an educational perspective, the findings suggest that a one-size-fits-all approach may not be effective. A blended learning model that leverages the strengths of both e-learning and face-to-face instruction could be optimal. Institutions should consider strategies to enhance student engagement in online settings, such as interactive discussions and collaborative projects, and provide faculty with training to effectively integrate technology into teaching. Furthermore, future intervention studies could evaluate the effectiveness of these strategies on both learning outcomes and students’ emotional well-being. Tracking changes in student learning over time in future studies could further inform best practices and the long-term impact of these interventions.

The study was conducted exclusively among undergraduate students, which may limit the generalizability of the findings to postgraduate populations. Future research should evaluate the applicability and measurement invariance of the ELFEQ across different levels of medical education.

## Supplementary Information

Below is the link to the electronic supplementary material.

Supplementary Material 1

## Data Availability

Yes, the data analyzed during the current study are available from the corresponding author on reasonable request.

## Abbreviations

F2F: Face-to-face
EFA: Exploratory Factor Analysis
CFA: Confirmatory Factor Analysis
ELFEQ: E-Learning and Face-to-Face Learning Experience Questionnaire

## Declarations

### AI use statement

During the preparation of this manuscript, AI-based tools were used to assist in the design of the figure. The authors reviewed and approved the final version and take full responsibility for the content.
