# Supplementary material for: Medical students’ perceptions of learning modalities: development and psychometric validation of the e-learning and face-to-face learning experience questionnaire
Source: Sci Rep. 2026 Jun 19;16:28162. doi: 10.1038/s41598-026-58815-1 (PMC13554172; doi:10.1038/s41598-026-58815-1)
Supplement: Supplementary file 1 [file 41598_2026_58815_MOESM1_ESM.docx]

**Comparative Questionnaire on the Effectiveness of Learning Tools in
Face-to-Face and Online Education from Students’ Perspectives**

**Dear Students,**

Greetings and best wishes for your health and academic success.

This questionnaire aims to compare the effectiveness of learning tools in face-to-face and online (virtual) education from students’ perspectives. Please answer the questions based on your own learning experiences.

We assure you that this questionnaire is anonymous, and your responses will be used solely for research purposes to evaluate learning tools and your experiences with online education.
Your honest and accurate responses will greatly assist university administrators in improving future educational planning.

**Demographic Information**

**Faculty:**
☐ Medicine  ☐ Paramedical Sciences  ☐ Management & Medical Informatics

☐ Dentistry  ☐ Rehabilitation  ☐ Modern Sciences

☐ Pharmacy  ☐ Public Health  ☐ Nursing  ☐ Nutrition  ☐ Other:

___________

**Field of Study:**

___________________________
**Year of Entry:** ___________________________

**Educational Level:**

☐ Bachelor’s degree ☐ Professional Doctorate ☐ Master’s degree ☐ PhD

**Age:** _________  **Gender:** ☐ Female ☐ Male

**Skill in Using Virtual Learning Systems (LMS):**

☐ Low  ☐ Moderate  ☐ I can solve almost all problems independently

**Access to Online Learning:**

Personal laptop or computer  ☐ Laptop/computer + mobile phone

☐ Mobile phone only  ☐ Internet café / other people’s computer

☐ Other: ___________________________

**Response Scale: 5 = Very High  4 = High  3 = Moderate  2 = Low  1 = Very Low**

Please rate each statement in **both columns** according to your experiences in
**previous face-to-face semesters** and **the recent online (virtual) semester.**

| **Components** | **Indicators** | **Face-to-Face** | **Online/Virtual** |
| --- | --- | --- | --- |
| Peer Assisted Learning | Q07. The discussions and conversations during the course were interesting and informative. | 1 – 2 – 3 – 4 - 5 | 1 – 2 – 3 – 4 - 5 |
|  | Q08. The discussions encouraged greater participation and engagement among students. | 1 – 2 – 3 – 4 - 5 | 1 – 2 – 3 – 4 - 5 |
|  | Q09. The discussions encouraged me to reflect on my own and my classmates’ opinions. | 1 – 2 – 3 – 4 - 5 | 1 – 2 – 3 – 4 - 5 |
|  | Q10. The discussions helped me learn from my classmates. | 1 – 2 – 3 – 4 - 5 | 1 – 2 – 3 – 4 - 5 |
|  | Q16. There was good interaction and question–answer exchange in the class. | 1 – 2 – 3 – 4 - 5 | 1 – 2 – 3 – 4 - 5 |
|  | Q17. I felt that my classmates played an effective role in my learning. | 1 – 2 – 3 – 4 - 5 | 1 – 2 – 3 – 4 - 5 |
| Teacher-Student Interaction | Q21. Seeing the instructor and having face-to-face interaction enhanced my learning. | 1 – 2 – 3 – 4 - 5 | 1 – 2 – 3 – 4 - 5 |
|  | Q22. Attending class sessions was important to me. | 1 – 2 – 3 – 4 - 5 | 1 – 2 – 3 – 4 - 5 |
|  | Q23. Courses were presented by instructors in an organized and sequential manner. | 1 – 2 – 3 – 4 - 5 | 1 – 2 – 3 – 4 - 5 |
|  | Q24. There was adequate interaction between instructors and students throughout the course. | 1 – 2 – 3 – 4 - 5 | 1 – 2 – 3 – 4 - 5 |
|  | Q25. There were opportunities for student participation during the course. | 1 – 2 – 3 – 4 - 5 | 1 – 2 – 3 – 4 - 5 |
|  | Q26. Instructors motivated students to study and learn. | 1 – 2 – 3 – 4 - 5 | 1 – 2 – 3 – 4 - 5 |
|  | Q27. I benefited from the instructors’ ethical behavior and role modeling. | 1 – 2 – 3 – 4 - 5 | 1 – 2 – 3 – 4 - 5 |
| Examination and Quizzes | Q11. Quizzes and exams were effective in reviewing and learning the course material. | 1 – 2 – 3 – 4 - 5 | 1 – 2 – 3 – 4 - 5 |
|  | Q12. Quizzes an Being comfortable d exams motivated me to study the course material. | 1 – 2 – 3 – 4 - 5 | 1 – 2 – 3 – 4 - 5 |
|  | Q13. Quizzes and exams helped me pay more attention to key points in the lessons. | 1 – 2 – 3 – 4 - 5 | 1 – 2 – 3 – 4 - 5 |
|  | Q14. The time allocated for answering quizzes was sufficient. | 1 – 2 – 3 – 4 - 5 | 1 – 2 – 3 – 4 - 5 |
|  | Q15. I completed the quizzes and exams myself without help from classmates. | 1 – 2 – 3 – 4 - 5 | 1 – 2 – 3 – 4 - 5 |
| Being comfortable | Q18. I could ask my questions freely in class without fear of judgment. | 1 – 2 – 3 – 4 - 5 | 1 – 2 – 3 – 4 - 5 |
|  | Q19. When the instructor asked questions, I could respond more comfortably. | 1 – 2 – 3 – 4 - 5 | 1 – 2 – 3 – 4 - 5 |
|  | Q20. During lessons, I was focused and less distracted by my surroundings. | 1 – 2 – 3 – 4 - 5 | 1 – 2 – 3 – 4 - 5 |
| Content | Q01. The scientific content of the courses was useful and informative (e.g., electronic materials, PowerPoints, etc.). | 1 – 2 – 3 – 4 - 5 | 1 – 2 – 3 – 4 - 5 |
|  | Q02. The educational content was well-designed both instructionally and audio visually. | 1 – 2 – 3 – 4 - 5 | 1 – 2 – 3 – 4 - 5 |
|  | Q03. The volume of course content provided during the semester was appropriate. | 1 – 2 – 3 – 4 - 5 | 1 – 2 – 3 – 4 - 5 |
| Assignments | Q04. I was able to access the course content and review or practice it at my own pace. | 1 – 2 – 3 – 4 - 5 | 1 – 2 – 3 – 4 - 5 |
|  | Q05. Assignments and exercises during the semester were effective in enhancing my learning. | 1 – 2 – 3 – 4 - 5 | 1 – 2 – 3 – 4 - 5 |
|  | Q06. Doing the assignments and learning activities encouraged me to study the course content. | 1 – 2 – 3 – 4 - 5 | 1 – 2 – 3 – 4 - 5 |
